# Coagulation factors and the incidence of COVID-19 severity: Mendelian randomization analyses and supporting evidence

**DOI:** 10.1038/s41392-021-00640-1

**Published:** 2021-06-07

**Authors:** Yao Zhou, Xinyi Qian, Zipeng Liu, Hongxi Yang, Tong Liu, Kexin Chen, Yaogang Wang, Pak Chung Sham, Ying Yu, Mulin Jun Li

**Affiliations:** 1 The Province and Ministry Co-sponsored Collaborative Innovation Center for Medical Epigenetics, Tianjin Medical University, Tianjin, China; 2 Centre for PanorOmic Sciences-Genomics and Bioinformatics Cores, The University of Hong Kong, Hong Kong, China; 3 National Clinical Research Center for Cancer, Tianjin Medical University Cancer Institute and Hospital, Tianjin Medical University, Tianjin, China

**Keywords:** Predictive markers, Genetics research, Genome informatics

**Dear Editor**,

The evolving pandemic of coronavirus disease 19 (COVID-19), is arousing alarm to public health. According to epidemiological and observational investigations, coagulopathy was frequently seen in severe COVID-19 patients^1^. Some coagulation factors such as D-dimer, prothrombin time (PT), von Willebrand factor (VWF), platelet count, and fibrinogen were documented to be important predictors of critically ill patients with COVID-19 in many retrospective observational studies and were substantially discussed before (see Supplementary Notes), yet the causality from specific coagulation factors to the incidence of COVID-19 severity and the underlying mechanism remains elusive.

To investigate the causal relationships between coagulation factors and the incidence of COVID-19 severity, we systematically curated genome-wide significant SNPs associated with 12 coagulation factors from different genome-wide association study (GWAS) results (Supplementary Table 1-2). After correlated instruments removal and effect size harmonization, we performed Mendelian Randomization (MR) analyses based on two largest GWASs of COVID-19 severity to date (Fig. 1a). In this process, several MR methodologies including Inverse variance weighted (IVW), MR-Egger regression, and weighted median (WM) methods were leveraged to test the causal effect of each coagulation factor on the incidence of COVID-19 severity, and various sensitivity analyses were applied to assess the robustness of our findings. As shown in Fig. 1b and Supplementary Table 3, our results revealed that genetic predisposition to the antigen levels of VWF and the activity levels of its cleaving protease, a disintegrin and metalloproteinase with a thrombospondin type 1 motif, member 13 (ADAMTS13) were causally associated with the incidence of COVID-19 severity.

**Fig. 1:**
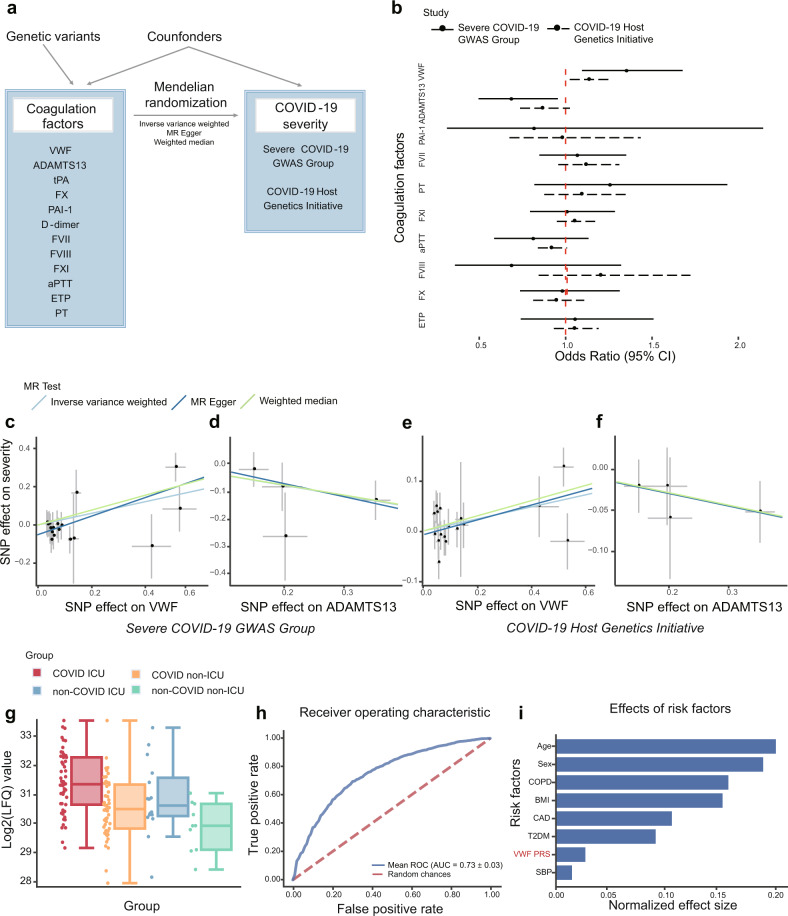
Mendelian randomization analyses and validation between coagulation factors and COVID-19 severity. **a** Mendelian randomization analysis framework in this study. A directed acyclic graph illustrates Mendelian randomization assumptions. The solid lines depict the potential causal diagram. **b** Forest plot shows odds ratio (OR) and 95% confidence interval (CI) from the results of IVW MR. The solid lines indicate MR results based on COVID-19 GWAS data from the Severe COVID-19 GWAS Group and the dashed lines indicate MR results based on COVID-19 GWAS data from the COVID-19 Host Genetics Initiative. D-dimer and tPA are excluded in this plot for abnormal OR values. **c**–**f** Scatter plots of the estimated genetic associations on the COVID-19 severity against the genetic association estimates with the VWF and ADAMTS13. The MR results are based on COVID-19 GWAS from (**c**, **d**) the Severe COVID-19 GWAS Group; (**e**, **f**) the COVID-19 Host Genetics Initiative. The slopes of the lines are the estimated causal effects using different MR methods including inverse variance weighted, MR Egger regression, and weighted median. **g** Relative abundance measurements of VWF protein in different patient groups. The relative abundance of VWF protein was estimated based on the relative abundances of its unique peptides. Different colors indicate patient status: COVID-19 ICU (red), COVID-19 non-ICU (orange), non-COVID-19 ICU (blue), and non-COVID-19 non-ICU (green). This image was created using data from COVID-19 Multi-Omics Data Dashboard (https://covid-omics.app). **h** Predictive ability of VWF PRS and clinical risk factors against COVID-19 severity. Receiver operating characteristic (ROC) for logistic regression using clinical risk factors and PRS derived from VWF GWAS as independent variables, the area under the receiver operating characteristic curve (AUC) was the mean value for 10-fold cross-validation. **i** Barplot depicts the normalized effect size of each contributing variable, values of each bar are coefficients of logistic regression after normalizing raw values to the same scale via z-score normalization. *MR* Mendelian randomization, *VWF* von Willebrand factor, *ADAMTS13* a disintegrin and metalloproteinase with a thrombospondin type 1 motif, member 13, *tPA* tissue plasminogen activator, *PAI-1* plasminogen activator inhibitor-1, *FVII* Factor VII, *PT* prothrombin time, *FVIII* Factor VIII, *FXI* Factor XI, aPTT activated partial thromboplastin time, *FX* Factor X, *ETP* endogenous thrombin potential, *LFQ* label-free quantification, *ICU* intensive care unit, *BMI* body mass index, *CAD* coronary artery disease, *COPD* chronic obstructive pulmonary disease, *PRS* polygenic risk score, *T2DM* type 2 diabetes mellitus

According to COVID-19 GWAS result from the Severe COVID-19 GWAS Group^2^, among all investigated coagulation factors, we observed that VWF (*P*_IVW_ = 0.005) and ADAMTS13 (*P*_IVW_ = 0.025) both showed significant results but displayed opposite direction of causal effect on the incidence of COVID-19 severity. Specifically, genetically determined plasma VWF antigen level was positively associated with the incidence of severe COVID-19 (*P*_IVW_ = 0.005, odds ratio (OR) = 1.35, 95% confidence interval (CI): 1.09–1.68, false discovery rate (FDR) = 0.06 (<10%)) based on 17 instrumental single-nucleotide polymorphisms (SNPs) (Fig. 1c). Both MR-Egger (*P*_Egger_ = 0.003) and WM MR (*P*_WM_ = 0.012) also supported the causal association (Fig. 1c and Supplementary Table 3). After removing the instruments that are significantly associated with confounder traits, no additional pleiotropy was detected between VWF levels and COVID-19 severity by Mendelian Randomization Pleiotropy RESidual Sum and Outlier (MR-PRESSO) global test (*P* = 0.074), Q_Egger_ (*P* = 0.777), and Q_IVW_ (*P* = 0.515). Besides, IVW MR revealed that plasma ADAMTS13 activity was inversely associated with the incidence of severe COVID-19 (*P*_IVW_ = 0.025, OR = 0.69, 95% CI: 0.50–0.96) based on four instrumental SNPs (Fig. 1d), and no pleiotropy was detected by MR-PRESSO global test (*P* = 0.772), Q_Egger_ (*P* = 0.433) or Q_IVW_ (*P* = 0.630). Interestingly, Given the VWF-cleaving function of ADAMTS13, this finding further supports the causal relationship between VWF levels and the incidence of COVID-19 severity. The statistical significance of ADAMTS13 disappeared after multiple testing correction (FDR = 0.15 (>10%)), which might be attributed to the relatively small number of valid instrumental variables.

In addition, based on COVID-19 severity GWAS data from the COVID-19 Host Genetics Initiative round 5^3^, we observed that VWF is the only coagulation factor that exhibited genetic causal associations with the incidence of COVID-19 severity (*P*_IVW_ = 0.029, OR = 1.13, 95% CI: 1.01–1.25, Fig. 1e). WM MR also revealed the significant causal association (*P*_WM_ = 0.046, OR = 1.16, 95% CI: 1.00–1.35, Fig. 1e and Supplementary Table 3). Sensitivity analyses supported the robustness of the result, where no pleiotropy was detected by MR-PRESSO global test (*P* = 0.104), Q_Egger_ (*P* = 0.245), and Q_IVW_ (*P* = 0.279). However, no significant signal was observed from the results of ADAMTS13 MR analyses (Fig. 1f). Taken together, these results confirmed that elevated VWF antigen level is a potential causal factor for the incidence of COVID-19 severity.

A growing body of studies reported that hypercoagulation status was frequently seen in COVID-19 patients^1^. We also performed a literature review to summarize existing clinical epidemiological studies regarding VWF/ADAMTS13 and COVID-19 severity. The majority of curated studies showed that the elevation of VWF antigen levels and the reduced ADAMTS13 activities are associated with COVID-19 severity (Supplementary Table 4). Besides, a multi-omics analysis leveraged RNA-Seq and high-resolution mass spectrometry on 128 blood samples from COVID-19 positive and negative patients with diverse disease severities, and found VWF antigen level is significantly higher in COVID-19 patients when compared to normal controls^4^. We further confirmed that the VWF protein level is significantly higher in intensive care unit (ICU) COVID-19 patients compared to non-ICU patients based on their released peptide quantifications (Fig. 1g). These evidences largely support that the antigen level of blood-derived VWF is an associated biomarker for COVID-19 severity.

Using an independent COVID-19 cohort from UK Biobank (UKBB), we identified 1492 severe COVID-19 cases and 445,271 healthy controls (baseline demographic and clinical characteristics are summarized in Supplementary Table 5). We explored the predictive ability of polygenic risk score (PRS) that derived from the VWF-associated genetic variants (17 instrumental SNPs) in the prediction of severe COVID-19 risk together with several critical risk factors, including age, sex, body mass index (BMI), coronary artery disease (CAD), systolic blood pressure (SBP), type 2 diabetes mellitus (T2DM), and chronic obstructive pulmonary disease (COPD)^5^. By evaluating the association of the VWF PRS and COVID-19 severity risk using a logistic regression model adjusted for the top 10 principal components of genetic variations and other selected risk factors (age, sex, BMI, CAD, SBP, T2DM, and COPD), we found that PRS of VWF is an independent risk factor for distinguishing severe COVID-19 cases from healthy controls, which explains a 16% higher risk (*P* = 0.011, OR per SD increase = 1.16, 95% CI: 1.03–1.29).

Furthermore, to investigate the prediction performance of the overall COVID-19 severity model and the contribution of VWF PRS, we calculated the area under the receiver operating characteristic curve (AUC) by 10-fold cross-validation. We found that the model combining clinical risk factors and the VWF PRS received a mean AUC of 0.734 (±0.03) (Fig. 1h), and the VWF PRS moderately increased the mean AUC by 0.3% when compared with the model based on only clinical parameters. Since we fitted the model with z-score normalized values, the coefficients of each contributing variable can be compared directly. We observed that age is the most important risk factor for COVID-19 severity, and male sex, high BMI, and history of COPD, CAD, and T2DM are also effective predictors (Fig. 1i), which is consistent with previous findings. Notably, VWF PRS showed a larger normalized effect size than SBP (Fig. 1i), emphasizing its predictive value during the prevention and personalized treatment of COVID-19.

In summary, together with the supporting evidence of recent retrospective cohort studies and independent validation based on UKBB data, our results suggest that the association between coagulation factor VWF and the incidence of COVID-19 severity is essentially causal, and the association between ADAMTS13 and the incidence of COVID-19 severity is likely to be causal, which illuminates one of the possible mechanisms underlying COVID-19 severity (Supplementary Notes). This study also highlights the importance of dynamically monitoring the plasma levels of VWF/ADAMTS13 after SARS-CoV-2 infection, and facilitates the development of a treatment strategy for controlling COVID-19 severity and associated thrombotic complication.

## Supplementary information

Supplementary Information

## Data Availability

All supporting data are included in the Supplementary Information.
