# Supplementary material for: Coagulation factors and the incidence of COVID-19 severity: Mendelian randomization analyses and supporting evidence
Source: Signal Transduct Target Ther. 2021 Jun 7;6:222. doi: 10.1038/s41392-021-00640-1 (PMC8181534; doi:10.1038/s41392-021-00640-1)
Supplement: Supplementary file 1 — Supplementary Information [file 41392_2021_640_MOESM1_ESM.docx]

**Supplementary Materials for**

**Coagulation factors and the incidence of COVID-19 severity: Mendelian randomization analyses and supporting evidence**

Yao Zhou, Xinyi Qian, Zipeng Liu, Hongxi Yang, Tong Liu, Kexin Chen, Yaogang Wang, Pak Chung Sham, Ying Yu, Mulin Jun Li^*^

Correspondence to: [mulinli@connect.hku.hk](mailto:mulinli@connect.hku.hk)

**This PDF file includes:**

Supplementary Methods

Supplementary Notes

Supplementary Tables S1 to S5

**Supplementary Methods**

Instrumental variables for coagulation factors

As summarized in Supplementary Table 2, we searched PubMed and GWAS Catalog ^1^ for the coagulation factor-relevant GWASs in European ethnic participants to identify genetic variants that could be used as instrumental variables for two-sample mendelian randomization (MR) analysis. Single-nucleotide polymorphisms (SNPs) associated with specific coagulation factor at sub-threshold genome-wide significant level (*P* < 5E-7) were selected as instrumental variables. The identified coagulation factors with genetic instruments include (1) Factor VIII (FVIII), Factor XI (FXI), and activated partial thromboplastin time (aPTT) that involved in intrinsic pathways; (2) Factor X (FX) and endogenous thrombin potential (ETP) that involved in common pathways; (3) Factor VII (FVII) and prothrombin time (PT) that involved in extrinsic pathways; (4) von Willebrand factor (VWF) and a thrombospondin type 1 motif, member 13 (ADAMTS13) that involved in platelet adhesion; (5) D-dimer, tissue plasminogen activator (tPA), and plasminogen activator inhibitor-1 (PAI-1) concentration that involved in the dissolution of fibrin clot. To ensure that MR results are more informative and horizontal pleiotropy can be assessed among instrumental variables, only coagulation factors with at least three genetic instruments were included in this study.

GWAS summary statistics for severe coronavirus disease 19 (COVID-19)

GWAS summary statistics for severe COVID-19 with respiratory failure were obtained from two sources, (1) the GWAS of severe COVID-19 with respiratory failure from the Severe COVID-19 GWAS Group ([http://www.c19-genetics.eu](http://www.c19-genetics.eu/)), which was based on 1,610 patients and 2,205 healthy control participants in European ethnic groups, wherein age, sex, and top 10 genetic components were adjusted ^2^; (2) the GWAS of COVID-19 with very severe respiratory from the COVID-19 Host Genetics Initiative ([https://www.covid19hg.org](https://www.covid19hg.org/), round 5, A2_ALL_leave_UKBB), which was based on 5,870 patients and 1,155,203 healthy control participants in mixed ethnic groups (mainly from European population) without UKBB cases ^3^.

UK Biobank (UKBB) COVID-19 data

The COVID-19 inspections result from the UKBB (up to 2021/3/1) was used, which included 446,763 unrelated (kinship coefficient > 0.0884, corresponding to 3rd-degree relationships) UKBB participants of European ancestry (mean age 70 years; 45.7% men). Specifically, we removed all participant without corresponding phenotype data, and then we directly included participants that do not relate to any other participants in our study, finally, we treated all participant pairs > KING kinship coefficient 0.0884 as candidate participants, we removed the individuals with the most relatedness to other participants in a stepwise manner, until there is no more relatedness among candidate participants, and included all remain participants to our study. All these participants were laboratory-confirmed COVID-19 patients. Baseline demographic and clinical characteristics of these participants are summarized in Supplementary Table 5.

The information of COVID-19 diagnosis was obtained from COVID-19 test results provided by Public Health England (PHE); death register provided by the National Health Service (NHS) Digital and NHS Central Register (NHSCR); hospital inpatient data provided by NHS Digital; and primary care data provided by TPP systems (https://www.tpp-uk.com/) and EMIS (<https://www.emishealth.com/>) systems. The selection criteria of UKBB participants included: ever reported as positive for SARS-CoV-2 by PHE; death from COVID-19 as the underlying cause (International Classification of Diseases, Tenth Revision (ICD-10): U071); hospitalization for COVID-19 (ICD-10: U071) from Hospital Episode Statistics; or confirmed COVID-19 infection from primary care data (Clinical Terms Version 3 (CTV3): Y20d1, EMIS: EMISNQCO303 or SNOMED CT: 1240581000000100).

Individual genotypes that primarily called by two genotyping arrays known as UKBB Axiom array and UK BiLEVE Axiom array were imputed to ~92 million autosomal and X-chromosome variants using merged panel comprised of UK10K haplotype reference panel and 1000 Genomes Phase 3 reference panel by UKBB.

Mendelian randomization instruments construction

The MR framework used in this study was shown in Fig. 1a. To ensure valid instruments selection according to MR analysis criteria ^4,5^, we first harmonized instrumental SNPs for each exposure GWAS by (1) excluding SNPs associated with other potential confounders (body mass index, lipid, and metabolic diseases, etc.) of exposure-outcome associations by searching PhenoScanner ^6^; (2) excluding SNPs located in the major histocompatibility complex (MHC) human leukocyte antigen (HLA) region from the list of instrumental SNPs due to potential horizontal pleiotropy with other confounder traits such as metabolic diseases and immune diseases ^7-9^; (3) excluding rare SNPs (minor allele frequency < 0.01) from the list of instrumental SNPs; (4) standardizing the effect size (β) and standard error (SE) for each SNP by the following formula ^10^:

$\beta=\frac{z}{\sqrt{2f(1-f)(n+z^{2})}}$ , $SE =\frac{1}{\sqrt{2f(1-f)(n+z^{2})}}$

where *z* = β/SE from the original summary data, *f* is the effect allele frequency, and *n* is the total sample size.

For SNPs that were not available in the GWAS data of COVID-19 severity, we used the LDlink tool ^11^ to find the most correlated proxies (r2 > 0.8), and the summary-level statistics for proxy SNPs were used instead. Based on SNPs reported to be associated with certain exposure by the above GWAS studies, we further applied the linkage disequilibrium (LD)-based clumping implemented in PLINK ^12^ (r^2^ threshold = 0.01 and window size = 10Mb) to ensure the independence of selected SNPs. Individual-level genotype data from European population of 1000 Genomes project served as the reference panel in this study. The final SNPs used as instruments in this study are summarized in Supplementary Table 1. To evaluate the strength of each instrument variable, F-statistics of instrumental variables were calculated with the following equation.

$\frac{R^{2}\left( n - 2 \right)}{(1 - R^{2})}$ ,

Where n is the sample size of the exposure and R^2^ is the amount of variance of the exposure explained by the SNP that computed as described by a previous study ^13^

MR statistical analysis

Several MR methodologies including Inverse variance weighted (IVW), MR-Egger regression, and weighted median (WM) methods were used to estimate the causal effects, wherein IVW was used in the main analysis ^14,15^. We obtained Wald ratio estimates for each instrumental SNP on COVID-19 severity, and then combined Wald ratio estimates using inverse variance weighting with fixed effects. To identify potential horizontal pleiotropy, we searched PhenoScanner ^6^ to explore whether instrumental SNPs are associated with other potential cofounders of exposure-outcome associations. Moreover, we further performed several sensitivity analyses to assess the robustness of our findings by (1) we evaluated heterogeneity for causal estimates that calculated by MR-Egger regression and IVW among instrument SNPs based on Cochran’s Q statistic ^16^ and then used Mendelian Randomization Pleiotropy RESidual Sum and Outlier (MR-PRESSO) global test ^17^ to detect global pleiotropy; (2) when the global test was significant (*P* < 0.05), we removed statistically significant outliers detected by MR-PRESSO outlier test (*P* < 0.05) and repeated MR analysis; (3) to avoid multiple comparisons problem, we applied Benjamini-Hochberg method to control False discovery rate (FDR significance threshold = 0.1), and the BH-adjusted *P*-values of IVW method were used in multiple comparisons correction. IVW, MR-Egger regression, WM methods were implemented in R package TwoSampleMR v0.5.4; MR-PRESSO global test and outlier test were implemented in R package MR-PRESSO.

Literature review

To gain more information regarding the two significant coagulation factors in MR analysis, we also performed a literature review to summarize existing clinical epidemiological studies regarding VWF/ADAMTS13 and COVID-19 severity. We used Google Scholar (up to 2020/10/1) to search terms: “COVID-19” or “SARS-CoV-2” or “2019-nCoV” or “Novel Coronavirus-Infected Pneumonia” or “2019 novel coronavirus” or “coronavirus 2019” and “severe” or “severity” and “VWF” or “Von Willebrand Factor” or “ADAMTS13” not only in the title and abstract, but also throughout the entire article.

Evaluation based on UKBB COVID-19 data

Severe COVID-19 cases from the UKBB cohort were defined as laboratory- or clinical- diagnosed COVID-19 patients with at least one of the following clinical features: (1) receiving care in the intensive care unit (ICU); (2) hospitalized inpatients; (3) depending on invasive ventilation using ventilator or ventilatory supports; (4) depending on noninvasive ventilation using other enabling machines and devices. Healthy controls were used for predicting the onset risk of severe COVID-19. We used PRSice-2 ^18^ to construct the PRS of VWF based on the effect sizes derived from the VWF GWASs and then investigated its association with the risk of critical illness in the COVID-19 patients from UKBB cohort. PRS based on significant instrumental SNPs of VWF were calculated by the following formula ^18^:

$$\mathrm{PRS}_{j}=\sum_{i} {\beta_{i}g}_{ij}$$

where *j* indicates *j*^th^ individual and *i* indicates *i*^th^ variant, g is the number of risk alleles carried ($g\in\left\{ 0,1,2 \right\}$), β is the harmonized effect size derived from the VWF GWASs.

We applied a multivariable logistic regression model to determine the association between the VWF PRS and the incidence of COVID-19 severity with adjustments for age, sex, body mass index (BMI), systolic blood pressure (SBP), top 10 principal components of genetic variations, history of coronary artery disease (CAD), type 2 diabetes mellitus (T2DM), and chronic obstructive pulmonary disease (COPD). The significance of regression coefficients was determined by Wald statistics test under null hypothesis that the variable has no correlation with COVID-19 severity risk. To demonstrate the predictive power of PRS, we trained two logistic regression models with (1) clinical risk factors and (2) clinical risk factors + VWF PRS. Before fitting the models, we applied z-score normalization to transform raw values of variables into a same scale. Due to the nature of a probabilistic statistical method, logistic regression models tend to be biased towards the majority class. To compensate the class imbalance issue between cases and controls, the probability weight based on inversed case-control ratio was used in the model ^19,20^, that is, the minor class was given more weight and the major class was given less weight (assigned 300:1 in this study). We used 10-fold cross-validation in the fitting process. Specifically, we randomly divided the entire sample into 10 equal-sized sub-samples, then iteratively fitted the model using the nine folds and validated the model using the remaining one fold. Furthermore, to evaluate the fit of the model, we took the mean value of an area under the receiver operating characteristic curve (AUC) measurement among the 10 iterations.

**Supplementary Notes**

Research background

The outbreak of COVID-19, which is caused by severe acute respiratory syndrome coronavirus 2 (SARS-CoV-2), with an unprecedented number of pneumonia cases from the late December 2019 put people on full alert ^21^. Widespread comorbidities implicating several organs were frequently observed in COVID-19 patients, such as diseases in cardiovascular, neurological, and hematopoietic systems ^22^. Of note, COVID-19-associated coagulopathy is a common complication among those patients developing severe systemic diseases and multiorgan failure ^23-25^, suggesting the importance of exploring clinical markers and the causal association between coagulopathy and COVID-19 ^26^.

Some coagulation parameters including D-dimer, prothrombin time (PT), von Willebrand factor (VWF), platelet count, and fibrinogen were previously documented to be important predictors of critically ill patients with COVID-19 ^27-29^. A recent study revealed that specific coagulation biomarkers, such as VWF and factor VIII (FVIII) levels, are independent predictors of increased oxygen requirements in COVID‐19 ^30^. It has also been observed that hospitalized COVID-19 patients, especially those with severe respiratory or systemic symptoms, are at increased risk for thromboembolism ^31,32^ and aberrant bleeding manifestations ^33,34^. Moreover, alveolar capillary microthrombi were 9 times as prevalent in patients who died from COVID-19 as those who died from H1N1 influenza ^35^. These retrospective observational studies clearly demonstrated the remarkable relevance among coagulation factor levels, thrombotic complications and COVID-19 severity. Nevertheless, it remains unclear which coagulation factor(s) can faithfully indicate the severity of COVID-19 illness or whether genetic predisposition to coagulation factor levels is causally related to severity and mortality of COVID-19 as well as the underlying biological pathways.

Novelty and clinical implication of this study

Emerging evidence from observational studies showed that COVID-19 patients are prone to developing thrombotic diseases ^36,37^ which are significantly associated with COVID-19 severity, poor prognosis, and mortality risk ^38,39^. However, whether exceptional plasma levels or activities of specific coagulation factors account for a higher risk of COVID-19 severity and aberrant thrombotic manifestation is elusive. In this study, we first explored the causal relationship between multiple coagulation factors and the incidence of COVID-19 severity using MR approaches. Together with the supporting evidence of recent retrospective cohort studies, our results revealed that the associations between VWF/ADAMTS13 and COVID-19 severity are essentially causal, suggesting the elevated VWF antigen and decreased ADAMTS13 activity are confident biomarkers that indicate progressive severity of COVID-19 and more aggressive critical care needed. In addition, genetic determents explain a considerable portion of the observed variance of plasma coagulation factor levels, including VWF levels ^40^. By PRS analysis on UKBB COVID-19 cohort, we uncovered that VWF PRS is an independent contributor for COVID-19 severity prediction. When combined with age, sex, BMI, and several pre-existing disease statuses, the model achieved good predictive ability in distinguishing severe cases from healthy controls.

VWF, stored in Weibel-Palade bodies and platelet α-granules for secretion upon stimulation, is a large multimeric glycoprotein which plays an important role in platelet recruitment after injury by forming a bridge between platelet surface receptors and endothelium ^41^. While, ADAMTS13 is a plasma protein cleaving VWF and decreasing its activity that anchored on the endothelial surface and in circulation ^42^. The dysfunction of VWF/ADAMTS13 dynamic equilibrium had been reported to be associated with thrombotic diseases ^43-46^ and cardiovascular diseases ^47^. COVID-19 patients are prone to developing thrombotic diseases ^36,37^, reciprocally, the development of thrombotic diseases could account for poor prognosis and mortality of COVID-19 ^38,39^. We suspected that SARS-COV-2 invades human lungs and causes injury or inflammation in the blood vessels, which then promoting a pro-coagulative state. As an important consideration for COVID-19, elevated VWF level and insufficient ADAMTS13 activity confer a higher risk of forming blood clots and ultimately develop venous thromboembolism. Thrombus can block normal blood flow and decrease oxygen supplement to alveoli, and this may explain partially why COVID-19 patients are at risk of respiratory failure. The patients with severe COVID-19 often have findings consistent with prominent endotheliopathy ^35,48,49^. The incidence of endotheliopathy and platelet activation are ubiquitous in COVID-19-associated coagulopathy and might play key roles in the progression of disease. Evidence showed that endotheliopathy could lead to the clinical prothrombotic manifestations of COVID-19-associated coagulopathy by augmented VWF release, platelet activation, and hypercoagulability ^29^. Mean level of VWF antigen in critical COVID-19 patients has been reported to range from 455 to more than 600% (normal range: 50-150%), mean level of ADAMTS13 activity in critical COVID-19 patients has been reported to range from 32.17 to 36% (normal range: 40-130%) ^29,50-54^. Regarding clinical practice, the association between VWF/ADAMTS13 and COVID-19 severity requires further confirmation, an ideal way to explore this association could be measuring VWF/ADAMTS13 at multiple time points during the hospitalization of COVID-19 patients and evaluate its correlation with the disease status. According to our MR causal inference and PRS analysis in the UKBB COVID-19 cohort, we could advise individuals carry certain VWF/ADAMTS13 alleles to closely monitor their coagulation factors and take supportive care for coagulopathy prevention after SARS-CoV-2 infection. Also, existing drugs targeting VWF or its molecular interactions could be considered to control COVID-19 severity and associated thrombotic complication for specific cohorts. Several other coagulation factors such as D-dimer and PT were previously documented to be associated with COVID-19 severity in observational studies ^27,28^, however, we found no causal relationships through MR analyses. This phenomenon could be attributed to potential confounding factors in observational studies, where associations between coagulation factors and COVID-19 severity was not due to direct causality, but rather because both coagulation factors and COVID-19 severity are likely caused by other unrevealed confounders.

Potential confounding factor and limitations

*ABO* gene loci obtained the most prominent GWAS signal in plasma VWF levels ^40^ and strong associations with COVID-19 ^2^, implying ABO blood group may confound the establishment of causality between VWF/ADAMTS13 and COVID-19 severity. Recent epidemiological studies have investigated and observed tight association of ABO blood group with the COVID-19 susceptibility, severity, and mortality ^55-58^. It was also reported that the *ABO* genotypes and ABO blood group are associated with ACE activity in hypertensive patients ^59^, and increased plasma ABO protein is causally associated with the risk of severe COVID-19 ^60^. Among the associated variants of VWF levels within the *ABO* locus, SNPs rs10901252 and rs687621 can perfectly discriminate B and O blood groups from A ^61^. Individuals with blood group O have lower VWF plasma concentrations compared with individuals with blood group non-O ^62^. The presence of blood group A and B antigens on VWF molecules may have clinically significant effects on VWF proteolysis and clearance ^63^. In our analysis, we excluded SNP rs687621 due to potential horizontal pleiotropy with other traits (such as Interleukin 6 levels and coronary artery disease) and rs10901252 for high LD with other instrumental SNPs (such as rs8176743). We found that the causality between VWF levels and COVID-19 severity was established when taking away the genetic effect of these SNPs. However, whether the causal mechanisms of VWF levels on COVID-19 severity could be independent of ABO blood group still needs further investigation.

Our study is also subject to some data limitations. First, only genome-wide significant variants were available from existing GWAS results of VWF/ADAMTS13 and most of the investigated coagulator factors, which makes it impossible to perform bi-directional MR analysis, and such limited number of instrumental SNPs for particular coagulation factors could affect the accuracy of both MR and PRS estimations. Similar to recent PRS studies on CAD ^64^ and ischemic stroke ^65^, we observed that incorporation of genetic component significantly contributes to the prediction model but only slightly improves the overall performance. The significant association between VWF PRS and COVID-19 severity indicated that the information captured by VWF PRS is not fully explained by other risk factors. But the current PRS study on COVID-19 cohort is likely underpowered due to insufficient sample size, the borderline significance required a larger study to ascertain the accuracy. Second, each method we utilized in MR analysis has its own assumptions, IVW obtains the causal estimate by a weighted regression of instrumental variable (IV) associations with an outcome on IV associations with an exposure ^66^. MR‐Egger attempts to model the distribution of the estimates from invalid IVs under the InSIDE (Instrument Strength Independent of Direct Effect) assumption ^67^. WM method is the median of a weighted empirical density function of the ratio estimates. WM method has been confirmed to have better finite‐sample Type 1 error rates than the IVW method and improved power of causal effect detection than MR‐Egger, but does not need InSIDE assumption ^68^. The usages of these methods may receive inconsistent results. Third, genetic risk loci for coagulation factors may vary among different populations ^69,70^, but our study mainly focuses on people of European descent. Whether there are population-specific causal mechanisms needs further exploration. Also, we didn’t investigate gender- or age-specific effects because of the lack of gender- or age-stratified GWAS data. Fourth, regarding the failure to replicate ADAMTS13 in the COVID-19 Host Genetics Initiative cohort, the major constraint could be relatively small number of valid instrumental variables on ADAMTS13, as well as some biases like improper severe case selection. It is worth noting that, the used GWAS from the COVID-19 Host Genetics Initiative is a multicenter meta-analysis compared to the result from the Severe COVID-19 GWAS Group, and it contains heterogeneous mixtures of sample populations and the between-study individual heterogeneity, which may obscure the inference of the causal effect ^71,72^. On the other hand, small number of instruments might lead to insufficient statistical power ^73^, thus our conclusion for ADAMTS13 remains conservative due to data limitation and needs more powerful analysis. Last, the sample size of severe COVID-19 GWAS is still insufficient in current stage, and further research is warranted when abundant and non-European ethnic GWAS data is available in the future. We also expect that prospective controlled trial could be applied to ascertain the causal role of VWF/ADAMTS13 and investigate potential treatments for certain infected populations.

**Supplementary Tables**

Table S1. Selected instrument SNPs of coagulation factors in this study

| **Exposure** | **SNP** | **Effect allele** | **Beta^a^** | **Se^b^** | ***P* value** | **F-statistics** | **Study** |
| --- | --- | --- | --- | --- | --- | --- | --- |
| VWF | rs55954186 | A | 0.04 | 0.01 | 8.715E-09 | 25.00 | Sabater-Lleal M ^61^ |
| VWF | rs548630 | A | -0.04 | 0.01 | 1.329E-12 | 36.00 |  |
| VWF | rs9390460 | T | -0.08 | 0.01 | 6.316E-38 | 120.99 |  |
| VWF | rs7788962 | A | -0.07 | 0.01 | 6.291E-08 | 21.78 |  |
| VWF | rs4276643 | T | 0.04 | 0.01 | 5.768E-27 | 93.44 |  |
| VWF | rs10985344 | A | 0.13 | 0.02 | 4.106E-09 | 32.11 |  |
| VWF | rs34434834 | A | 0.12 | 0.01 | 6.348E-12 | 42.97 |  |
| VWF | rs2238109 | A | 0.09 | 0.01 | 1.37E-82 | 277.76 |  |
| VWF | rs4981022 | A | 0.05 | 0.01 | 5.442E-37 | 136.10 |  |
| VWF | rs4904820 | A | -0.05 | 0.01 | 8.301E-18 | 53.78 |  |
| VWF | rs6494314 | T | -0.06 | 0.01 | 9.628E-08 | 36.00 |  |
| VWF | rs2277998 | A | -0.05 | 0.01 | 2.024E-15 | 58.77 |  |
| VWF | rs5750823 | T | -0.42 | 0.07 | 2.978E-11 | 40.11 |  |
| VWF | rs8176743 | C | -0.51 | 0.04 | 1.59E-17 | 33.46 | Williams ^74^ |
| VWF | rs12518614 | A | -0.53 | 0.06 | 1.52E-09 | 184.78 |  |
| VWF | rs651007 | C | -0.14 | 0.02 | 4.415E-36 | 69.06 |  |
| VWF | rs216321 | T | 0.04 | 0.01 | 1.7E-17 | 61.92 | Tang W ^75^ |
| ADAMTS13 | rs10456544 | A | 0.20 | 0.04 | 1.1E-08 | 29.39 | de Vries PS ^76^ |
| ADAMTS13 | rs4075970 | G | 0.15 | 0.02 | 6.8E-09 | 35.98 | Ma Q ^77^ |
| ADAMTS13 | rs28673647 | G | 0.35 | 0.02 | 1.2E-57 | 201.52 |  |
| ADAMTS13 | rs3124762 | C | 0.20 | 0.03 | 8.9E-09 | 34.01 |  |
| PAI-1 | rs2227631 | A | 0.08 | 0.01 | 7.80E-15 | 63.99 | Huang J ^78^ |
| PAI-1 | rs3847067 | A | 0.07 | 0.01 | 4.10E-10 | 36.00 |  |
| PAI-1 | rs6486122 | T | 0.05 | 0.01 | 3.00E-08 | 25.00 |  |
| PAI-1 | rs314376 | G | 0.05 | 0.01 | 2.40E-09 | 25.00 |  |
| PAI-1 | rs11128603 | A | 0.07 | 0.02 | 2.90E-08 | 20.25 |  |
| ETP | rs35800856 | A | 1.11 | 0.12 | 6.13E-09 | 80.92 | Rocanin-Arjo A ^79^ |
| ETP | rs17787912 | G | -0.28 | 0.04 | 1.63E-11 | 53.72 |  |
| ETP | rs2856656 | C | 0.38 | 0.06 | 4.62E-22 | 37.17 |  |
| D-Dimer | rs12029080 | G | 0.053 | 0.011 | 6.00E-52 | 25.00 | Smith NL ^80^ |
| D-Dimer | rs6687813 | A | 0.056 | 0.021 | 2.00E-14 | 7.37 |  |
| D-Dimer | rs13109457 | A | 0.034 | 0.011 | 3.00E-18 | 9.00 |  |
| tPA | rs9399599 | T | 0.06 | 0.01 | 2.90E-14 | 56.25 | Huang J ^81^ |
| tPA | rs3136739 | A | 0.12 | 0.02 | 1.30E-09 | 36.00 |  |
| tPA | rs7301826 | C | 0.07 | 0.01 | 1.00E-09 | 56.25 |  |
| FVII | rs569557 | G | 0.65 | 0.01 | 6.4E-600 | 2738.58 | de Vries PS ^82^ |
| FVII | rs867186 | G | 0.26 | 0.01 | 3.3E-64 | 360.97 |  |
| FVII | rs1260326 | T | 0.10 | 0.01 | 2.3E-30 | 143.99 |  |
| FVII | rs7935829 | G | 0.08 | 0.01 | 6.3E-18 | 80.99 |  |
| FVII | rs6532796 | G | 0.07 | 0.01 | 2.6E-13 | 64.00 |  |
| FVII | rs1149616 | T | 0.06 | 0.01 | 1.7E-10 | 32.11 |  |
| FVII | rs10761784 | A | 0.06 | 0.01 | 6.7E-10 | 42.25 |  |
| FVII | rs498475 | G | 0.05 | 0.01 | 1.5E-08 | 36.00 |  |
| PT | rs6027 | T | 0.19 | 0.02 | 5.90E-29 | 128.44 |  |
| PT | rs7901813 | A | 0.05 | 0.01 | 6.50E-10 | 38.10 |  |
| PT | rs4399232 | T | -0.06 | 0.01 | 9.30E-13 | 51.29 |  |
| PT | rs491098 | C | -0.32 | 0.01 | 2.80E-147 | 695.00 |  |
| PT | rs1801690 | C | -0.13 | 0.02 | 3.30E-13 | 56.25 |  |
| PT | rs2069940 | C | -0.15 | 0.01 | 5.20E-29 | 115.97 |  |
| FXI | rs710446 | T | -0.40 | 0.01 | 2.07E-302 | 1380.31 | Sennblad B ^83^ |
| FXI | rs4253417 | T | -0.33 | 0.01 | 2.86E-193 | 878.25 |  |
| FXI | rs780094 | T | 0.07 | 0.01 | 3.56E-09 | 34.85 |  |
| aPTT | rs2239852 | T | 0.09 | 0.02 | 1.90E-08 | 35.99 | Weng L ^84^ |
| aPTT | rs710446 | C | -0.41 | 0.01 | 2.30E-197 | 870.07 |  |
| aPTT | rs9898 | T | -0.36 | 0.01 | 1.20E-123 | 600.13 |  |
| aPTT | rs4253399 | G | -0.16 | 0.01 | 1.40E-25 | 115.54 |  |
| aPTT | rs1801020 | A | 0.61 | 0.02 | 8.30E-260 | 1501.25 |  |
| aPTT | rs657152 | A | -0.27 | 0.01 | 5.00E-75 | 351.49 |  |
| FVIII | rs548630 | A | -0.05 | 0.01 | 5.14E-10 | 36.00 | Sabater-Lleal M ^61^ |
| FVIII | rs9399599 | A | -0.06 | 0.01 | 3.73E-13 | 49.00 |  |
| FVIII | rs7816579 | A | 0.07 | 0.01 | 5.32E-16 | 68.65 |  |
| FVIII | rs10102164 | A | 0.05 | 0.01 | 1.66E-07 | 23.90 |  |
| FVIII | rs35165583 | G | -0.07 | 0.01 | 1.74E-10 | 41.75 |  |
| FVIII | rs7135039 | T | 0.08 | 0.01 | 3.00E-19 | 83.59 |  |
| FVIII | rs4981022 | A | 0.07 | 0.01 | 1.16E-17 | 64.00 |  |
| FVIII | rs137631 | T | 0.06 | 0.01 | 2.35E-07 | 24.10 |  |
| FVIII | rs698078 | A | 0.06 | 0.01 | 4.26E-07 | 27.42 |  |
| FVIII | rs7962217 | C | 0.13 | 0.02 | 6.30E-09 | 34.00 | Tang W ^75^ |
| FVIII | rs12557310 | C | -0.07 | 0.01 | 8.02E-10 | 37.51 |  |
| FX | rs547138 | A | 0.23 | 0.02 | 8.91E-20 | 82.98 | Sun BB ^85^ |
| FX | rs141217364 | A | -0.29 | 0.03 | 2.57E-19 | 80.64 |  |
| FX | rs3762056 | T | -0.54 | 0.04 | 3.02E-34 | 149.12 |  |

^a,b^ Beta and Se are standardized for each SNP by the following formula ^10^:

$\beta=\frac{z}{\sqrt{2f(1-f)(n+z^{2})}}$ , $se =\frac{1}{\sqrt{2f(1-f)(n+z^{2})}}$

where z = β/se from the original summary data, f is the effect allele frequency, and n is the total sample size.

**Abbreviations: VWF**, von Willebrand factor; **ADAMTS13**, a disintegrin and metalloproteinase with a thrombospondin type 1 motif, member 13; **tPA**, tissue plasminogen activator; **PAI-1**, plasminogen activator inhibitor-1; **FVII**, Factor VII; **PT**, prothrombin time; **FVIII**, Factor VIII; **FXI**, Factor XI; **aPTT**, activated partial thromboplastin time; **FX**, Factor X; **ETP**, endogenous thrombin potential.

Table S2. Data sources for 12 investigated coagulation factors in this study

| **Pathway^a^** | **Phenotype** | **Sample Size** | **Study** | **PMID** | **Consortium/Study** |
| --- | --- | --- | --- | --- | --- |
| Platelet adhesion to exposed collagen | VWF | 42,256 | Sabater-Lleal M ^61^ | 30586737 | CHARGE |
|  |  | 2,100 | Williams ^74^ | 23381943 | TwinsUK |
|  |  | 18,556 | Tang W ^75^ | 25779970 | CARe |
|  | ADAMTS13 | 5,448 | de Vries PS ^76^ | 25934476 | Rotterdam |
|  |  | 3,238 | Ma Q ^77^ | 29296746 | GABC + TSS |
| Dissolution of fibrin clot | D-dimer | 21,052 | Smith NL ^80^ | 21502573 | CHARGE |
|  | PAI-1 | 19,599 | Huang J ^78^ | 22990020 | FHS + PROCARDIS +TwinsUK + MONICA/KORA + HealthABC + MARTHA + PREVEND |
|  | tPA | 19,599 | Huang J ^81^ | 24578379 | CHARGE |
| Extrinsic pathway | FVII | 27,495 | de Vries PS ^82^ | 30642921 | CHARGE |
|  | PT | 30,972 | Goldstein JA ^86^ | \ | BioVU + MGI |
| Intrinsic pathway | FXI | 16,169 | Sennblad B ^83^ | 28053049 | ARIC + PROCARDIS + GHS-1 + GHS-2 + MARTHA |
|  | aPTT | 9,719 | Weng L ^84^ | 25552651 | ARIC |
|  | FVIII | 29,573 | Sabater-Lleal M ^61^ | 30586737 | CHARGE |
|  |  | 18,556 | Tang W ^75^ | 25779970 | CARe |
| Common pathway | FX | 3,301 | Sun BB ^85^ | 29875488 | INTERVAL |
|  | ETP | 1,967 | Rocanin-Arjo A ^79^ | 24357727 | MARTHA + 3C |

**Abbreviations:** **CHARGE**, Cohorts for Heart and Aging Research in Genomic Epidemiology; **GABC**, Genes and Blood Clotting; **TSS**, Trinity Student Study; **FHS**, Framingham HeartStudy; **PROCARDIS**, Precocious Coronary Artery Disease Study; **MARTHA**, Marseille Thrombosis Association study; **PREVEND**, Prevention of Renal and Vascular End Stage; Disease study; **MGI**, Michigan Genomics Initiative; **ARIC**, Atherosclerosis Risk in Communities; **CARe**, Candidate Gene Association Resource; **INTERVAL**, International Network against VENous Thrombosis; **3C**, Three Cities Study.

**VWF**, von Willebrand factor; **ADAMTS13**, a disintegrin and metalloproteinase with a thrombospondin type 1 motif, member 13; **tPA**, tissue plasminogen activator; **PAI-1**, plasminogen activator inhibitor-1; **FVII**, Factor VII; **PT**, prothrombin time; **FVIII**, Factor VIII; **FXI**, Factor XI; **aPTT**, activated partial thromboplastin time; **FX**, Factor X; **ETP**, endogenous thrombin potential.

^a^ Pathways are classified based on the Reactome database ^87^ by Harshfield EL ^88^.

Table S3. Summary statistics of the MR estimates of coagulation factors on COVID-19 severity

| **Exposure** | **nSNPs** | **IVW OR (95% CI)** | ***P*_IVW_** | **Egger OR (95% CI)** | ***P*_Egger_** | **Egger**  **Intercept** | **Egger**  **Intercept *P*** | **WM OR (95% CI)** | ***P*_WM_** | **IVW OR (95% CI)** | ***P*_IVW_** | **Egger OR (95% CI)** | ***P*_Egger_** | **Egger**  **Intercept** | **Egger**  **Intercept *P*** | **WM OR (95% CI)** | ***P*_WM_** |
| --- | --- | --- | --- | --- | --- | --- | --- | --- | --- | --- | --- | --- | --- | --- | --- | --- | --- |
|  |  | The Severe COVID-19 GWAS Group ^2^ | | | | | | | | The COVID-19 Host Genetics Initiative ^3^ | | | | | | | |
| VWF | 17 | 1.35  (1.09-1.68) | **0.005** | 1.62  (1.24-2.13) | **0.003** | -0.05 | 0.513 | 1.47  (1.09-2.00) | **0.012** | 1.13  (1.01-1.25) | **0.029** | 1.16  (1.01-1.33) | 0.055 | -0.01 | 0.533 | 1.16  (1.00-1.35) | **0.046** |
| ADAMTS13 | 4 | 0.69  (0.50-0.96) | **0.025** | 0.62  (0.25-1.53) | 0.409 | 0.03 | 0.829 | 0.69  (0.48-0.99) | **0.044** | 0.86  (0.73-1.03) | 0.099 | 0.85  (0.54-1.36) | 0.626 | 0.01 | 0.963 | 0.86  (0.72-1.03) | 0.099 |
| D-dimer | 3 | 3.80  (0.52-27.90) | 0.189 | 133.44  (0.01-2203234.91) | 0.504 | -0.17 | 0.597 | 3.66  (0.28-47.28) | 0.320 | 0.88  (0.28-2.80) | 0.831 | 41.73  (0.88-1981.36) | 0.309 | -0.18 | 0.295 | 1.27  (0.45-3.57) | 0.654 |
| PAI-1 | 5 | 0.82  (0.31-2.14) | 0.685 | 0.06  (0.01-9.19) | 0.348 | 0.18 | 0.370 | 0.58  (0.17-1.92) | 0.371 | 0.97  (0.66-1.42) | 0.883 | 0.82  (0.10-6.39) | 0.859 | 0.01 | 0.876 | 0.93  (0.59-1.47) | 0.761 |
| tPA | 3 | 1.46  (0.15-14.27) | 0.743 | 1.51  (0.01-752.29) | 0.918 | -0.29 | 0.438 | 0.88  (0.14-5.75) | 0.897 | 1.11  (0.68-1.84) | 0.671 | 1.86  (0.12-28.98) | 0.734 | -0.04 | 0.773 | 1.35  (0.75-2.45) | 0.316 |
| FVII | 8 | 1.07  (0.85-1.35) | 0.569 | 0.95  (0.70-1.28) | 0.742 | 0.04 | 0.253 | 1.04  (0.81-1.34) | 0.769 | 1.11  (0.95-1.30) | 0.202 | 1.02  (0.84-1.25) | 0.814 | 0.03 | 0.260 | 1.08  (0.97-1.21) | 0.167 |
| PT | 6 | 1.26  (0.82-1.94) | 0.294 | 1.04 (0.51-2.11) | 0.917 | 0.04 | 0.540 | 1.13  (0.69-1.83) | 0.631 | 1.08  (0.86-1.36) | 0.482 | 1.05  (0.70-1.59) | 0.816 | 0.01 | 0.872 | 1.16  (0.90-1.48) | 0.256 |
| FXI | 3 | 1.01  (0.79-1.29) | 0.934 | 0.96  (0.59-1.58) | 0.904 | 0.02 | 0.862 | 1.01  (0.78-1.29) | 0.958 | 1.04  (0.94-1.16) | 0.437 | 1.05  (0.82-1.33) | 0.781 | 0.01 | 0.987 | 1.04  (0.94-1.16) | 0.445 |
| aPTT | 6 | 0.81  (0.59-1.13) | 0.223 | 1.08  (0.52-2.23) | 0.849 | -0.11 | 0.441 | 0.98  (0.80-1.20) | 0.839 | 0.91  (0.83-1.00) | 0.045 | 0.85  (0.69-1.05) | 0.214 | 0.02 | 0.542 | 0.92  (0.85-1.00) | 0.047 |
| FVIII | 11 | 0.69  (0.36-1.32) | 0.262 | 0.68  (0.03-17.22) | 0.819 | 0.01 | 0.992 | 0.95  (0.41-2.22) | 0.913 | 1.20  (0.84-1.71) | 0.328 | 1.09  (0.21-5.64) | 0.917 | 0.01 | 0.916 | 1.12  (0.71-1.77) | 0.633 |
| FX | 3 | 0.98  (0.74-1.31) | 0.908 | 1.57  (0.70-3.50) | 0.472 | -0.16 | 0.439 | 0.93  (0.67-1.30) | 0.676 | 0.94  (0.80-1.10) | 0.416 | 1.04  (0.66-1.65) | 0.896 | -0.03 | 0.719 | 0.94  (0.79-1.11) | 0.455 |
| ETP | 3 | 1.06  (0.74-1.51) | 0.761 | 1.05  (0.40-2.75) | 0.937 | 0.01 | 0.990 | 0.98  (0.73-1.31) | 0.881 | 1.04  (0.92-1.18) | 0.514 | 1.11  (0.88-1.40) | 0.544 | -0.04 | 0.647 | 1.05  (0.91-1.20) | 0.516 |

Values in bold indicate statistically significant results.

**Abbreviations: VWF**, von Willebrand factor; **ADAMTS13**, a disintegrin and metalloproteinase with a thrombospondin type 1 motif, member 13; **tPA**, tissue plasminogen activator; **PAI-1**, plasminogen activator inhibitor-1; **FVII**, Factor VII; **PT**, prothrombin time; **FVIII**, Factor VIII; **FXI**, Factor XI; **aPTT**, activated partial thromboplastin time; **FX**, Factor X; **ETP**, endogenous thrombin potential.

Table S4. Supporting evidence for the associations between VWF/ADAMTS13 activities and COVID-19 severity

| **Study** | **PMID** | **Area** | **Sample size** | **Criteria for severity** | **Findings** |
| --- | --- | --- | --- | --- | --- |
| Helms J ^52^ | 32367170 | French | 150 | Admitted to ICU | VWF antigen level was increased in patients with severe COVID-19 compared to healthy controls |
| Panigada M ^89^ | 32302438 | Italy | 24 | Admitted to ICU because of acute respiratory syndromes | VWF antigen level was considerably increased in ICU patients compared to healthy controls |
| Bazzan M ^54^ | 32557383 | United States | 88 | Death | Patients who died had significant lower levels of ADAMTS13 and higher levels of VWF compared to patients with non-fatal outcome |
| Krishnamachary B ^90^ | 32909001 | United States | 53 | Requires oxygen delivery by non-rebreather mask, non-invasive ventilation, or heated high flow nasal cannula at a minimum | Significantly elevated levels of VWF and decreased ADAMTS13 in large extracellular vesicles from patients with severe COVID-19 compared to healthy controls |
| Overmyer KA ^91^ | 32743614 | United States | 128 | Admitted to ICU | VWF level was increased in ICU patients versus in non-ICU patients |
| Adam EH ^53^ | 32723615 | Germany | 4 | Admitted to ICU | Elevated levels of VWF and decreased ADAMTS13 in ICU patients |
| Adrian AN ^92^ | medRxiv | Germany | 85 | The severity degree of COVID-19 was categorized according to the guidelines of the Robert Koch Institute, Germany | Elevated levels of VWF and decreased ADAMTS13/VWF ratio in severe patients |
| Goshua, G ^29^ | 32619411 | United States | 68 | Admitted to ICU | VWF antigen level was significantly elevated in ICU patients compared with non-ICU patients |

**Abbreviations:** **ICU**, intensive care unit; **VWF**, von Willebrand factor; **ADAMTS13**, a disintegrin and metalloproteinase with a thrombospondin type 1 motif, member 13

Table S5. Baseline demographic and clinical characteristics of UK Biobank COVID-19 cohort

| **Characteristics** | **Case**  **(n = 1,492)** | **Controls**  **(n = 445,271)** | | ***P* value** |
| --- | --- | --- | --- | --- |
| Age | 72 ± 8 | | 70 ± 8 | < 0.001 |
| Male sex | 900 (60) | | 203,326 (46) | < 0.001 |
| Body mass index (kg/m^2^) | 30 ± 5 | | 27 ± 5 | < 0.001 |
| Systolic blood pressure (mmHg) | 142 ± 19 | | 139 ± 19 | < 0.001 |
| Comorbidities |  | |  |  |
| Coronary artery disease | 410 (27) | | 42,849 (9) | < 0.001 |
| Type 2 diabetes | 381 (26) | | 31,367 (7) | < 0.001 |
| COPD | 295 (18) | | 20,293 (5) | < 0.001 |

Values are n (%) or mean ± SD.

**Reference**

1 Buniello, A. *et al.* The NHGRI-EBI GWAS Catalog of published genome-wide association studies, targeted arrays and summary statistics 2019. *Nucleic acids research* **47**, D1005-D1012, doi:10.1093/nar/gky1120 (2019).

2 Severe Covid, G. G. *et al.* Genomewide Association Study of Severe Covid-19 with Respiratory Failure. *N Engl J Med* **383**, 1522-1534, doi:10.1056/NEJMoa2020283 (2020).

3 Initiative, C.-H. G. The COVID-19 Host Genetics Initiative, a global initiative to elucidate the role of host genetic factors in susceptibility and severity of the SARS-CoV-2 virus pandemic. *Eur J Hum Genet* **28**, 715-718, doi:10.1038/s41431-020-0636-6 (2020).

4 Burgess, S. *et al.* Using published data in Mendelian randomization: a blueprint for efficient identification of causal risk factors. *Eur J Epidemiol* **30**, 543-552, doi:10.1007/s10654-015-0011-z (2015).

5 Zhang, K. *et al.* Causally Associations of Blood Lipids Levels with COVID-19 Risk: Mendelian Randomization Study. *medRxiv*, 2020.2007.2007.20147926, doi:10.1101/2020.07.07.20147926 (2020).

6 Kamat, M. A. *et al.* PhenoScanner V2: an expanded tool for searching human genotype-phenotype associations. *Bioinformatics* **35**, 4851-4853, doi:10.1093/bioinformatics/btz469 (2019).

7 Matzaraki, V., Kumar, V., Wijmenga, C. & Zhernakova, A. The MHC locus and genetic susceptibility to autoimmune and infectious diseases. *Genome Biol* **18**, 76, doi:10.1186/s13059-017-1207-1 (2017).

8 Wellcome Trust Case Control, C. Genome-wide association study of 14,000 cases of seven common diseases and 3,000 shared controls. *Nature* **447**, 661-678, doi:10.1038/nature05911 (2007).

9 Tomer, Y. *et al.* Genome wide identification of new genes and pathways in patients with both autoimmune thyroiditis and type 1 diabetes. *J Autoimmun* **60**, 32-39, doi:10.1016/j.jaut.2015.03.006 (2015).

10 Zhu, Z. *et al.* Integration of summary data from GWAS and eQTL studies predicts complex trait gene targets. *Nat Genet* **48**, 481-487, doi:10.1038/ng.3538 (2016).

11 Machiela, M. J. & Chanock, S. J. LDlink: a web-based application for exploring population-specific haplotype structure and linking correlated alleles of possible functional variants. *Bioinformatics* **31**, 3555-3557, doi:10.1093/bioinformatics/btv402 (2015).

12 Chang, C. C. *et al.* Second-generation PLINK: rising to the challenge of larger and richer datasets. *GigaScience* **4**, 7, doi:10.1186/s13742-015-0047-8 (2015).

13 Burgess, S., Davies, N. M. & Thompson, S. G. Bias due to participant overlap in two-sample Mendelian randomization. *Genet Epidemiol* **40**, 597-608, doi:10.1002/gepi.21998 (2016).

14 Swerdlow, D. I. *et al.* Selecting instruments for Mendelian randomization in the wake of genome-wide association studies. *Int J Epidemiol* **45**, 1600-1616, doi:10.1093/ije/dyw088 (2016).

15 Grover, S. Mendelian Randomization: Methods for Using Genetic Variants in Causal Estimation. S.Burgess and S. G.Thompson (2015). London, UK: Chapman & Hall/CRC Press. 224 pages, ISBN: 9781466573178. *Biometrical Journal* **59**, 1086-1087, doi:10.1002/bimj.201700051 (2017).

16 Bowden, J. *et al.* A framework for the investigation of pleiotropy in two-sample summary data Mendelian randomization. *Stat Med* **36**, 1783-1802, doi:10.1002/sim.7221 (2017).

17 Verbanck, M., Chen, C. Y., Neale, B. & Do, R. Detection of widespread horizontal pleiotropy in causal relationships inferred from Mendelian randomization between complex traits and diseases. *Nat Genet* **50**, 693-698, doi:10.1038/s41588-018-0099-7 (2018).

18 Choi, S. W. & O'Reilly, P. F. PRSice-2: Polygenic Risk Score software for biobank-scale data. *GigaScience* **8**, doi:10.1093/gigascience/giz082 (2019).

19 King, G. & Zeng, L. Logistic Regression in Rare Events Data. *Political Analysis* **9**, 137-163, doi:10.1093/oxfordjournals.pan.a004868 (2017).

20 Maalouf, M. & Siddiqi, M. Weighted logistic regression for large-scale imbalanced and rare events data. *Knowl-Based Syst* **59**, 142-148, doi:10.1016/j.knosys.2014.01.012 (2014).

21 Zhu, N. *et al.* A Novel Coronavirus from Patients with Pneumonia in China, 2019. *N Engl J Med* **382**, 727-733, doi:10.1056/NEJMoa2001017 (2020).

22 Richardson, S. *et al.* Presenting Characteristics, Comorbidities, and Outcomes Among 5700 Patients Hospitalized With COVID-19 in the New York City Area. *JAMA* **323**, 2052-2059, doi:10.1001/jama.2020.6775 (2020).

23 Zhou, F. *et al.* Clinical course and risk factors for mortality of adult inpatients with COVID-19 in Wuhan, China: a retrospective cohort study. *Lancet* **395**, 1054-1062, doi:10.1016/S0140-6736(20)30566-3 (2020).

24 Becker, R. C. COVID-19 update: Covid-19-associated coagulopathy. *J Thromb Thrombolysis* **50**, 54-67, doi:10.1007/s11239-020-02134-3 (2020).

25 Ortega-Paz, L., Capodanno, D., Montalescot, G. & Angiolillo, D. J. COVID-19 Associated Thrombosis and Coagulopathy: Review of the Pathophysiology and Implications for Antithrombotic Management. *J Am Heart Assoc* **0**, e019650, doi:10.1161/JAHA.120.019650 (2020).

26 The Lancet, H. COVID-19 coagulopathy: an evolving story. *Lancet Haematol* **7**, e425, doi:10.1016/S2352-3026(20)30151-4 (2020).

27 Liao, D. *et al.* Haematological characteristics and risk factors in the classification and prognosis evaluation of COVID-19: a retrospective cohort study. *Lancet Haematol* **7**, e671-e678, doi:10.1016/S2352-3026(20)30217-9 (2020).

28 Tang, N., Li, D., Wang, X. & Sun, Z. Abnormal coagulation parameters are associated with poor prognosis in patients with novel coronavirus pneumonia. *J Thromb Haemost* **18**, 844-847, doi:10.1111/jth.14768 (2020).

29 Goshua, G. *et al.* Endotheliopathy in COVID-19-associated coagulopathy: evidence from a single-centre, cross-sectional study. *Lancet Haematol* **7**, e575-e582, doi:10.1016/S2352-3026(20)30216-7 (2020).

30 Rauch, A. *et al.* Coagulation biomarkers are independent predictors of increased oxygen requirements in COVID-19. *Journal of Thrombosis and Haemostasis* **18**, 2942-2953, doi:<https://doi.org/10.1111/jth.15067> (2020).

31 Giannis, D., Ziogas, I. A. & Gianni, P. Coagulation disorders in coronavirus infected patients: COVID-19, SARS-CoV-1, MERS-CoV and lessons from the past. *J Clin Virol* **127**, 104362, doi:10.1016/j.jcv.2020.104362 (2020).

32 Connors, J. M. & Levy, J. H. COVID-19 and its implications for thrombosis and anticoagulation. *Blood* **135**, 2033-2040, doi:10.1182/blood.2020006000 (2020).

33 Chan, N. C. & Weitz, J. I. COVID-19 coagulopathy, thrombosis, and bleeding. *Blood* **136**, 381-383, doi:10.1182/blood.2020007335 (2020).

34 Al-Samkari, H. *et al.* COVID-19 and coagulation: bleeding and thrombotic manifestations of SARS-CoV-2 infection. *Blood* **136**, 489-500, doi:10.1182/blood.2020006520 (2020).

35 Ackermann, M. *et al.* Pulmonary Vascular Endothelialitis, Thrombosis, and Angiogenesis in Covid-19. *N Engl J Med* **383**, 120-128, doi:10.1056/NEJMoa2015432 (2020).

36 Middeldorp, S. *et al.* Incidence of venous thromboembolism in hospitalized patients with COVID-19. *J Thromb Haemost* **18**, 1995-2002, doi:10.1111/jth.14888 (2020).

37 Poissy, J. *et al.* Pulmonary Embolism in Patients With COVID-19: Awareness of an Increased Prevalence. *Circulation* **142**, 184-186, doi:10.1161/CIRCULATIONAHA.120.047430 (2020).

38 Zhang, L. *et al.* Deep Vein Thrombosis in Hospitalized Patients With COVID-19 in Wuhan, China. *Circulation* **142**, 114-128, doi:10.1161/CIRCULATIONAHA.120.046702 (2020).

39 Bilaloglu, S. *et al.* Thrombosis in Hospitalized Patients With COVID-19 in a New York City Health System. *JAMA* **324**, 799-801, doi:10.1001/jama.2020.13372 (2020).

40 Desch, K. C. Regulation of plasma von Willebrand factor. *F1000Res* **7**, 96, doi:10.12688/f1000research.13056.1 (2018).

41 Bryckaert, M., Rosa, J. P., Denis, C. V. & Lenting, P. J. Of von Willebrand factor and platelets. *Cell Mol Life Sci* **72**, 307-326, doi:10.1007/s00018-014-1743-8 (2015).

42 Dong, J. F. *et al.* ADAMTS-13 rapidly cleaves newly secreted ultralarge von Willebrand factor multimers on the endothelial surface under flowing conditions. *Blood* **100**, 4033-4039, doi:10.1182/blood-2002-05-1401 (2002).

43 Koster, T., Blann, A. D., Briet, E., Vandenbroucke, J. P. & Rosendaal, F. R. Role of clotting factor VIII in effect of von Willebrand factor on occurrence of deep-vein thrombosis. *Lancet* **345**, 152-155, doi:10.1016/s0140-6736(95)90166-3 (1995).

44 Sadler, J. E. Von Willebrand factor, ADAMTS13, and thrombotic thrombocytopenic purpura. *Blood* **112**, 11-18, doi:10.1182/blood-2008-02-078170 (2008).

45 Obermeier, H. L. *et al.* The role of ADAMTS-13 and von Willebrand factor in cancer patients: Results from the Vienna Cancer and Thrombosis Study. *Res Pract Thromb Haemost* **3**, 503-514, doi:10.1002/rth2.12197 (2019).

46 Pepin, M. *et al.* ADAMTS-13 and von Willebrand factor predict venous thromboembolism in patients with cancer. *J Thromb Haemost* **14**, 306-315, doi:10.1111/jth.13205 (2016).

47 Matsukawa, M. *et al.* Serial changes in von Willebrand factor-cleaving protease (ADAMTS13) and prognosis after acute myocardial infarction. *Am J Cardiol* **100**, 758-763, doi:10.1016/j.amjcard.2007.03.095 (2007).

48 Gu, S. X. *et al.* Thrombocytopathy and endotheliopathy: crucial contributors to COVID-19 thromboinflammation. *Nat Rev Cardiol* **18**, 194-209, doi:10.1038/s41569-020-00469-1 (2021).

49 Rauch, A. *et al.* Endotheliopathy Is Induced by Plasma From Critically Ill Patients and Associated With Organ Failure in Severe COVID-19. *Circulation* **142**, 1881-1884, doi:10.1161/Circulationaha.120.050907 (2020).

50 Philippe, A. *et al.* Circulating Von Willebrand factor and high molecular weight multimers as markers of endothelial injury predict COVID-19 in-hospital mortality. *Angiogenesis*, doi:10.1007/s10456-020-09762-6 (2021).

51 Escher, R., Breakey, N. & Lammle, B. Severe COVID-19 infection associated with endothelial activation. *Thromb Res* **190**, 62, doi:10.1016/j.thromres.2020.04.014 (2020).

52 Helms, J. *et al.* High risk of thrombosis in patients with severe SARS-CoV-2 infection: a multicenter prospective cohort study. *Intensive Care Med* **46**, 1089-1098, doi:10.1007/s00134-020-06062-x (2020).

53 Adam, E. H., Zacharowski, K. & Miesbach, W. A comprehensive assessment of the coagulation profile in critically ill COVID-19 patients. *Thromb Res* **194**, 42-44, doi:10.1016/j.thromres.2020.06.026 (2020).

54 Bazzan, M. *et al.* Low ADAMTS 13 plasma levels are predictors of mortality in COVID-19 patients. *Intern Emerg Med* **15**, 861-863, doi:10.1007/s11739-020-02394-0 (2020).

55 Zhao, J. *et al.* Relationship between the ABO Blood Group and the COVID-19 Susceptibility. *Clin Infect Dis*, doi:10.1093/cid/ciaa1150 (2020).

56 Zeng, X. *et al.* Association between ABO blood groups and clinical outcome of coronavirus disease 2019: Evidence from two cohorts. *medRxiv*, 2020.2004.2015.20063107, doi:10.1101/2020.04.15.20063107 (2020).

57 Rao, S., Lau, A. & So, H. C. Exploring Diseases/Traits and Blood Proteins Causally Related to Expression of ACE2, the Putative Receptor of SARS-CoV-2: A Mendelian Randomization Analysis Highlights Tentative Relevance of Diabetes-Related Traits. *Diabetes Care* **43**, 1416-1426, doi:10.2337/dc20-0643 (2020).

58 Latz, C. A. *et al.* Blood type and outcomes in patients with COVID-19. *Ann Hematol* **99**, 2113-2118, doi:10.1007/s00277-020-04169-1 (2020).

59 Chung, C. M. *et al.* A genome-wide association study identifies new loci for ACE activity: potential implications for response to ACE inhibitor. *The Pharmacogenomics Journal* **10**, 537-544, doi:10.1038/tpj.2009.70 (2010).

60 Hernandez Cordero, A. I. *et al.* Multi-omics highlights ABO plasma protein as a causal risk factor for COVID-19. 2020.2010.2005.20207118, doi:10.1101/2020.10.05.20207118 %J medRxiv (2020).

61 Sabater-Lleal, M. *et al.* Genome-Wide Association Transethnic Meta-Analyses Identifies Novel Associations Regulating Coagulation Factor VIII and von Willebrand Factor Plasma Levels. *Circulation* **139**, 620-635, doi:10.1161/CIRCULATIONAHA.118.034532 (2019).

62 Franchini, M., Capra, F., Targher, G., Montagnana, M. & Lippi, G. Relationship between ABO blood group and von Willebrand factor levels: from biology to clinical implications. *Thromb J* **5**, 14, doi:10.1186/1477-9560-5-14 (2007).

63 Jenkins, P. V. & O'Donnell, J. S. ABO blood group determines plasma von Willebrand factor levels: a biologic function after all? *Transfusion* **46**, 1836-1844, doi:10.1111/j.1537-2995.2006.00975.x (2006).

64 Elliott, J. *et al.* Predictive Accuracy of a Polygenic Risk Score–Enhanced Prediction Model vs a Clinical Risk Score for Coronary Artery Disease. *JAMA* **323**, 636-645, doi:10.1001/jama.2019.22241 (2020).

65 Abraham, G. *et al.* Genomic risk score offers predictive performance comparable to clinical risk factors for ischaemic stroke. *Nature Communications* **10**, 5819, doi:10.1038/s41467-019-13848-1 (2019).

66 Burgess, S., Butterworth, A. & Thompson, S. G. Mendelian Randomization Analysis With Multiple Genetic Variants Using Summarized Data. *Genet Epidemiol* **37**, 658-665, doi:10.1002/gepi.21758 (2013).

67 Slob, E. A. W. & Burgess, S. A comparison of robust Mendelian randomization methods using summary data. *Genet Epidemiol* **44**, 313-329, doi:10.1002/gepi.22295 (2020).

68 Bowden, J., Smith, G. D., Haycock, P. C. & Burgess, S. Consistent Estimation in Mendelian Randomization with Some Invalid Instruments Using a Weighted Median Estimator. *Genet Epidemiol* **40**, 304-314, doi:10.1002/gepi.21965 (2016).

69 Miller, C. H., Dilley, A., Richardson, L., Hooper, W. C. & Evatt, B. L. Population differences in von Willebrand factor levels affect the diagnosis of von Willebrand disease in African-American women. *Am J Hematol* **67**, 125-129, doi:10.1002/ajh.1090 (2001).

70 Sukhu, K., Poovalingam, V., Mahomed, R. & Giangrande, P. L. Ethnic variation in von Willebrand factor levels can influence the diagnosis of von Willebrand disease. *Clin Lab Haematol* **25**, 247-249, doi:10.1046/j.1365-2257.2003.00523.x (2003).

71 Xie, Y. Population heterogeneity and causal inference. *Proc Natl Acad Sci U S A* **110**, 6262-6268, doi:10.1073/pnas.1303102110 (2013).

72 Zheng, J. *et al.* Recent Developments in Mendelian Randomization Studies. *Curr Epidemiol Rep* **4**, 330-345, doi:10.1007/s40471-017-0128-6 (2017).

73 Pierce, B. L., Ahsan, H. & VanderWeele, T. J. Power and instrument strength requirements for Mendelian randomization studies using multiple genetic variants. *International Journal of Epidemiology* **40**, 740-752, doi:10.1093/ije/dyq151 (2011).

74 Williams, F. M. *et al.* Ischemic stroke is associated with the ABO locus: the EuroCLOT study. *Ann Neurol* **73**, 16-31, doi:10.1002/ana.23838 (2013).

75 Tang, W. *et al.* Gene-centric approach identifies new and known loci for FVIII activity and VWF antigen levels in European Americans and African Americans. *Am J Hematol* **90**, 534-540, doi:10.1002/ajh.24005 (2015).

76 de Vries, P. S. *et al.* Genetic variants in the ADAMTS13 and SUPT3H genes are associated with ADAMTS13 activity. *Blood* **125**, 3949-3955, doi:10.1182/blood-2015-02-629865 (2015).

77 Ma, Q. *et al.* Genetic variants in ADAMTS13 as well as smoking are major determinants of plasma ADAMTS13 levels. *Blood Adv* **1**, 1037-1046, doi:10.1182/bloodadvances.2017005629 (2017).

78 Huang, J. *et al.* Genome-wide association study for circulating levels of PAI-1 provides novel insights into its regulation. *Blood* **120**, 4873-4881, doi:10.1182/blood-2012-06-436188 (2012).

79 Rocanin-Arjo, A. *et al.* A meta-analysis of genome-wide association studies identifies ORM1 as a novel gene controlling thrombin generation potential. *Blood* **123**, 777-785, doi:10.1182/blood-2013-10-529628 (2014).

80 Smith, N. L. *et al.* Genetic predictors of fibrin D-dimer levels in healthy adults. *Circulation* **123**, 1864-1872, doi:10.1161/CIRCULATIONAHA.110.009480 (2011).

81 Huang, J. *et al.* Genome-wide association study for circulating tissue plasminogen activator levels and functional follow-up implicates endothelial STXBP5 and STX2. *Arterioscler Thromb Vasc Biol* **34**, 1093-1101, doi:10.1161/ATVBAHA.113.302088 (2014).

82 de Vries, P. S. *et al.* A genome-wide association study identifies new loci for factor VII and implicates factor VII in ischemic stroke etiology. *Blood* **133**, 967-977, doi:10.1182/blood-2018-05-849240 (2019).

83 Sennblad, B. *et al.* Genome-wide association study with additional genetic and post-transcriptional analyses reveals novel regulators of plasma factor XI levels. *Human molecular genetics* **26**, 637-649, doi:10.1093/hmg/ddw401 (2017).

84 Weng, L. C. *et al.* A genetic association study of activated partial thromboplastin time in European Americans and African Americans: the ARIC Study. *Human molecular genetics* **24**, 2401-2408, doi:10.1093/hmg/ddu732 (2015).

85 Sun, B. B. *et al.* Genomic atlas of the human plasma proteome. *Nature* **558**, 73-79, doi:10.1038/s41586-018-0175-2 (2018).

86 Goldstein, J. A. *et al.* LabWAS: Novel findings and study design recommendations from a meta-analysis of clinical labs in two independent biobanks. *PLoS Genet* **16**, e1009077, doi:10.1371/journal.pgen.1009077 (2020).

87 Jassal, B. *et al.* The reactome pathway knowledgebase. *Nucleic acids research* **48**, D498-D503, doi:10.1093/nar/gkz1031 (2020).

88 Harshfield, E. L., Sims, M. C., Traylor, M., Ouwehand, W. H. & Markus, H. S. The role of haematological traits in risk of ischaemic stroke and its subtypes. *Brain* **143**, 210-221, doi:10.1093/brain/awz362 (2020).

89 Panigada, M. *et al.* Hypercoagulability of COVID-19 patients in intensive care unit: A report of thromboelastography findings and other parameters of hemostasis. *J Thromb Haemost* **18**, 1738-1742, doi:10.1111/jth.14850 (2020).

90 Krishnamachary, B., Cook, C., Spikes, L., Chalise, P. & Dhillon, N. K. The Potential Role of Extracellular Vesicles in COVID-19 Associated Endothelial injury and Pro-inflammation. *medRxiv*, 2020.2008.2027.20182808, doi:10.1101/2020.08.27.20182808 (2020).

91 Overmyer, K. A. *et al.* Large-Scale Multi-omic Analysis of COVID-19 Severity. *Cell Syst* **12**, 23-40 e27, doi:10.1016/j.cels.2020.10.003 (2021).

92 Doevelaar, A. A. N. *et al.* COVID-19 is associated with relative ADAMTS13 deficiency and VWF multimer formation resembling TTP. *medRxiv*, 2020.2008.2023.20177824, doi:10.1101/2020.08.23.20177824 (2020).
